# Increased Serum Levels of Macrophage Inflammatory Protein-3α and Cystatin A Predict a Poor Prognosis of Nasopharyngeal Carcinoma

**DOI:** 10.1097/MD.0000000000000123

**Published:** 2014-11-07

**Authors:** Yonglin Cai, Jun Li, Aiying Lu, Weiming Zhong, Jianquan Gao, Yuming Zheng, Hong Zeng, Wei Wang, Minzhong Tang

**Affiliations:** Wuzhou Health System Key Laboratory for Nasopharyngeal Carcinoma Etiology and Molecular Mechanism (YC, JL, AL, YZ, HZ, MT); Clinical Laboratory (YC, JL, AL); Department of Radiation Oncology (WZ, JG), Wuzhou Red Cross Hospital, Wuzhou, Guangxi; Second People’s Hospital of Zhuhai (WW), Zhuhai, Guangdong; and College of Life Science and Bioengineering (MT), Beijing University of Technology, Beijing, China.

## Abstract

This study was aimed to investigate the roles of serum macrophage inflammatory protein-3α (MIP-3α) and cystatin A in nasopharyngeal carcinoma (NPC) prognosis.

The serum levels of MIP-3α and cystatin A in 140 primary NPC patients without distant metastasis were detected by enzyme-linked immunosorbent assay before and after treatment. The results were compared with those in 100 healthy controls. The log-rank test was used to compare survival curves of the 2 groups. Multivariate analysis of prognostic factors used Cox proportional hazards regression model.

Serum levels of MIP-3α and cystatin A in pretreatment patients with NPC were higher than those in healthy controls. Concentrations of these 2 factors in the majority of patients after the therapy decreased to control level. Patients with high serum level of MIP-3α and cystatin A before treatment had poorer overall survival (OS), local recurrence-free survival, and distant metastasis-free survival than the ones with low level. In addition, serum pretreatment MIP-3α and cystatin A levels were independent prognostic factors for OS and distant metastasis-free survival of NPC patients; serum posttreatment MIP-3α and cystatin A levels were independent prognostic factors of local recurrence-free survival.

Our results revealed that serum MIP-3α and cystatin A may be promising candidate prognostic factors for NPC, and higher serum levels of MIP-3α and cystatin A correlate with shorter probability of OS, local recurrence, and distant metastasis.

## INTRODUCTION

Nasopharyngeal carcinoma (NPC) constitutes a serious health problem in southern China as it is one of the most common cancers there. Wuzhou City of Guangxi province in southern China is one of the endemic areas with high NPC incidence.^1–3^ NPC is sensitive to radiotherapy, and the 5-year overall survival (OS) and progression-free survival rates for advanced NPC treated with concurrent chemoradiotherapy were about 70%.^4,5^ The prognostic prediction of NPC is mainly based on clinical TNM staging nowadays. However, the fact that patients with the same clinical stage have various therapeutic effects indicates that TNM stage does not accurately predict the prognosis for NPC. So far, it is mostly dependent on imaging examination for checking NPC residual lesion, relapse, and distant metastasis, evaluating the sensitivity of radiotherapy and chemotherapy, and predicting prognosis. Identification of serum tumor markers of NPC is urgently needed and may be helpful for convenient and noninvasive early disease screening, prognosis prediction, treatment planning, and residual or recurrent tumor monitoring.

An important pathological feature of NPC is a wide range of lymphocytic infiltration. Although the characteristics of tumor infiltrating lymphocytes have not yet been elucidated, interaction between tumor cells and tumor-infiltrating lymphocytes in NPC microenvironment may play an important role in tumor formation, and cytokines may mediate the interaction as well. Chemokines are recently reported to be involved in tumor proliferation, progression, and metastasis.^6^ Chemokines are classified into 4 families (CC, CXC, CX_3_C, and XC) based on the number and spacing of cysteine residues and exert their biological effects by interacting with specific cell surface receptors that belong to transmembrane G protein-coupled receptor family. These chemokines can activate the genes associated with cell movement, invasion, and metastasis through different transmembrane signal pathways. Macrophage inflammatory protein-3α (MIP-3α), encoded by the *CCL20* gene, is a chemokine that induces leukocyte migration into inflammation sites and regulates leukocyte trafficking through lymphoid tissues; and its expression has effects on tumor cell proliferation, angiogenesis, inflammatory cell infiltration, invasion, and metastasis.^7^ Lysosomal cysteine proteases (cathepsins) may degrade extracellular matrix, which is a key step for tumor cells breaking through the basement membrane and then causing invasion and metastasis. So far, more and more reports suggest that cystatin families, as the endogenous inhibitors of cathepsins, are closely associated with tumorigenesis, development, invasion, and metastasis, and it may be a novel tumor marker for clinical diagnosis and prognostic prediction.^8^

MIP-3α and cystatin A have been reported as novel biomarkers and prognosis predictors for NPC.^9,10^ The clinical significance of serum MIP-3α and cystatin A levels at different time points (pretreatment vs posttreatment) has not been fully explored. In this prospective study, we evaluated the serum MIP-3α and cystatin A levels before and after treatment in NPC and correlated the results with treatment outcome.

## PATIENTS AND METHODS

### Study Population

From February 2009 to May 2010, a total of 140 NPC patients, who were previously untreated, biopsy-proven, with no evidence of distant metastasis, and hospitalized in Wuzhou Red Cross Hospital, were included prospectively in this study. All patients underwent routine check-ups, including clinical examinations of the head and neck region, fiber optic nasopharyngoscopy, head and neck magnetic resonance imaging, chest x-rays, abdominal ultrasonography, and emission computed tomography. Keratinizing squamous cell carcinoma was classified as World Health Organization (WHO) type I, nonkeratinizing differentiated carcinoma as type II, and nonkeratinizing undifferentiated carcinoma as type III.^11^ The cancer stage was defined according to the Chinese 2008 staging system^12^ (Table 1). Clinical information of the patients is described in detail in Table 2. There were 101 male patients and 39 female patients, with a male-to-female ratio of 2.6:1; their median age was 47 years, with a range of 10 to 76 years.

**TABLE 1:**
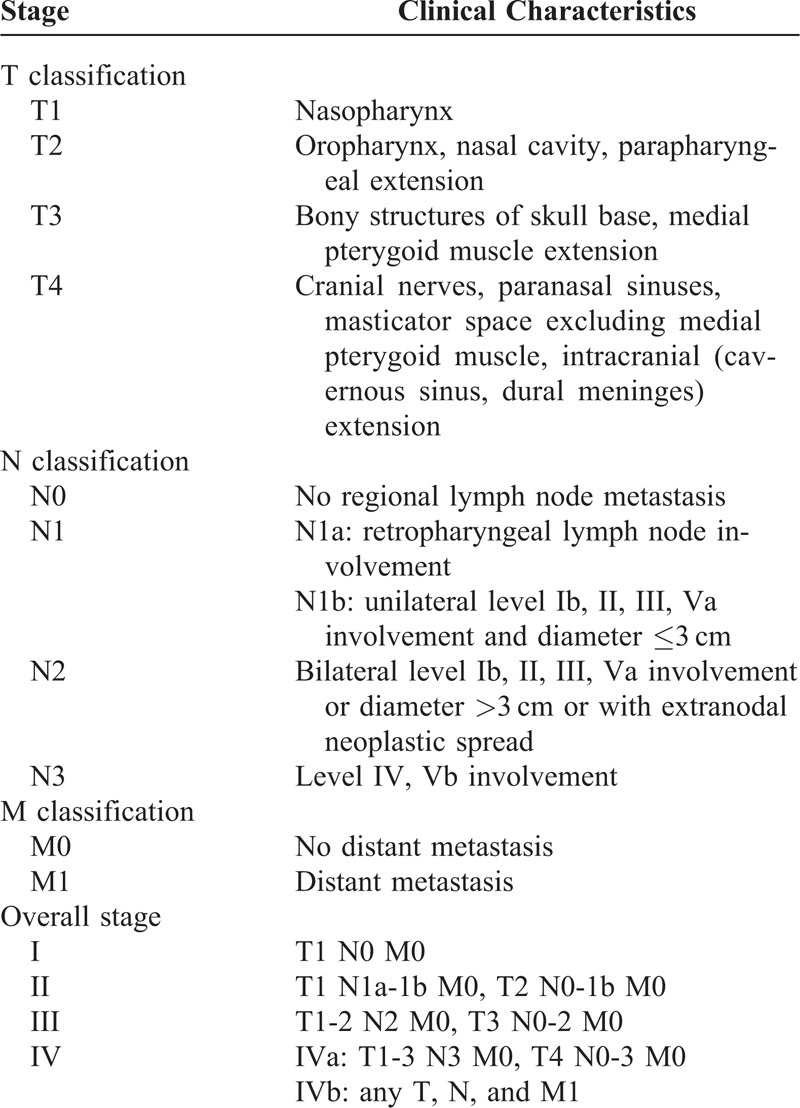
Chinese 2008 Staging System for Nasopharyngeal Carcinoma

**TABLE 2:**
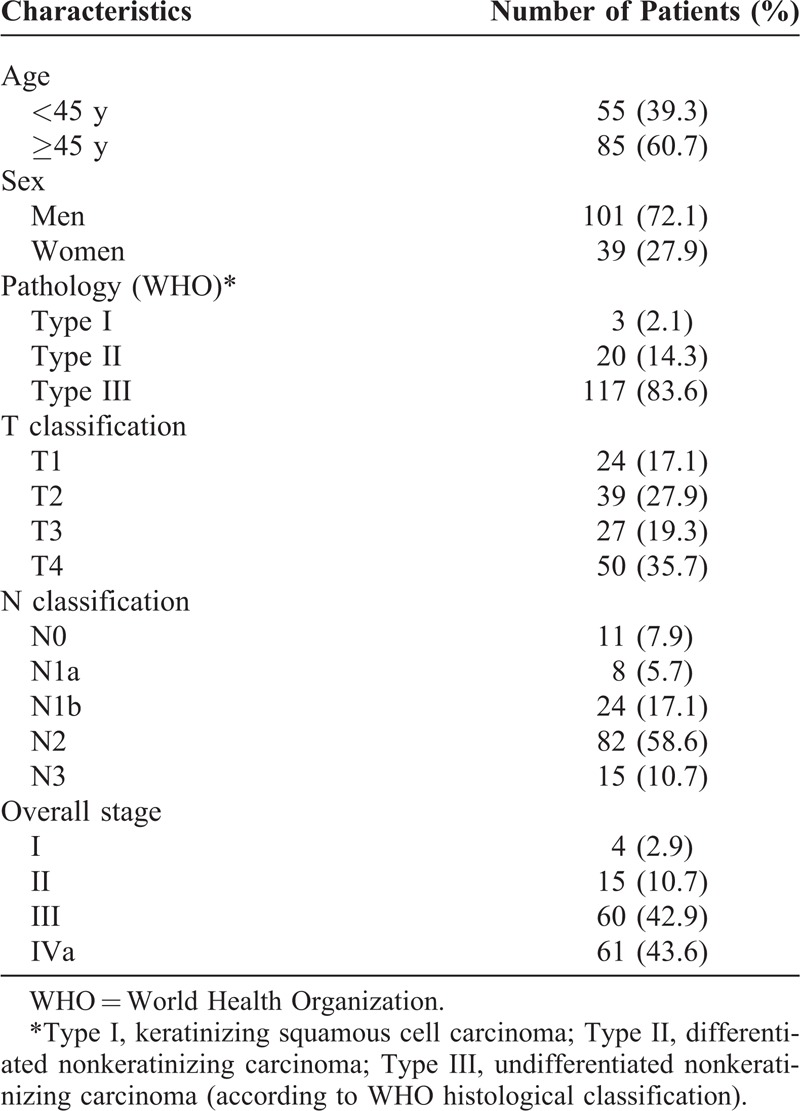
Clinical Characteristics and Tumor Parameters of 140 Patients With Nasopharyngeal Carcinoma

All patients underwent radical radiotherapy for NPC. For patients with stages I–II, the treatment was radiotherapy alone, whereas for those with stages III–IVa, chemotherapy was added to radiotherapy. Two radiotherapy technologies were used, including conventional 2-dimensional radiotherapy and 3-dimensional conformal radiotherapy. The accumulated doses to gross primary tumor and involved neck lymph nodes were 68 to 74 Gy and 64 to 70 Gy, respectively; the uninvolved areas received 50 to 56 Gy. Chemotherapy consisted of cisplatin and 5-fluorouracil. The chemotherapy regimen of cisplatin 25 mg/m^2^ was delivered over 3 days for 3 cycles on d1, d22, and d43 during the course of radiotherapy as concurrent chemotherapy. Subsequent adjuvant chemotherapy consisted of 25 mg/m^2^ and 5-fluorouracil 450 mg/m^2^ injected on days 1 to 3 for 2 courses. Peripheral blood was taken before the commencement of treatment and after the completion of treatment.

Blood samples without any evidence of disease were obtained from 100 healthy individuals as controls. There were 71 male participants and 29 female participants; their median age was 49 years, with a range of 21 to 75 years. The clinical research ethics committee of Wuzhou Red Cross Hospital approved this study.

### Enzyme-Linked Immunosorbent Assay for MIP-3α and Cystatin A

Sera from peripheral blood samples were obtained by venipuncture at study enrollment and stored at −70°C until use. Serum levels of MIP-3α (expressed in picogram/milliliter) and cystatin A (expressed in nanogram/milliliter) were determined using a commercially available human MIP-3α or cystatin A quantitative Enzyme-Linked Immunosorbent Assay kit (USCN, Wuhan, China) according to the manufacturer’s instructions. All standards and samples were prepared and tested in duplicate and the mean value taken. The levels of MIP-3α or cystatin A were separately calculated from the standard curves.

### Statistical Analysis

The OS was calculated from the first day of chemoradiotherapy until the date of death or until the date of the last follow-up. The local recurrence-free survival (LRFS) was calculated from the first day of chemoradiotherapy until the date of either primary lesions in the nasopharynx or regional lymph node recurrence or until the date of the last follow-up. The distant metastasis-free survival (DMFS) was calculated from the first day of chemoradiotherapy until the date of distant metastasis or until the date of the last follow-up.

The levels of serum MIP-3α and cystatin A in different groups were compared using the Mann–Whitney rank sum test. Survival curves were plotted by the Kaplan–Meier method and compared by the log-rank test. The significance of various variables for survival was analyzed by the Cox proportional hazards regression model. Variables that were included in the model were age, sex, pathological type, tumor (T)-classification, lymph node (N)-classification, and chemotherapy along with MIP-3α and cystatin A levels before and after treatment. All statistical analyses were performed using SPSS version 13.0 (SPSS Inc, Chicago, IL), and all *P* values were 2-sided and values <0.05 were considered as statistically significant.

## RESULTS

The end of follow-up was January 26, 2014. After a median follow-up of 48 months (range, 5–61 months), 9 patients were lost to follow-up, 11 patients were reported with local recurrence, 16 with distant metastasis, 5 with local recurrence plus distant metastasis, and 25 deaths. The 4-year follow-up rate was 93.6% (131/140). The 4-year OS, LRFS, and DMFS rates for all patients were 84.9%, 88.6%, and 84.7%, respectively.

We found that serum levels of MIP-3α and cystatin A in pretreatment patients with NPC were higher than those in healthy controls; the majority of patients had decreased levels of MIP-3α and cystatin A during the therapy. The mean values of decreases were 63.62 pg/mL and 7.10 ng/mL for MIP-3α and cystatin A, respectively. There was no statistical significance for MIP-3α and cystatin A levels between posttreatment patients and healthy controls (*P* = 0.252 and 0.850) (Table 3).

**TABLE 3:**
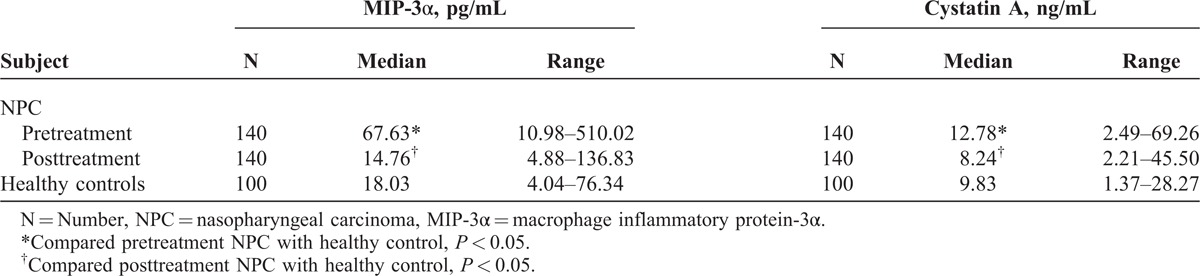
Serum Levels of MIP-3α and Cystatin A in Pretreatment Patients, Posttreatment Patients With NPC, and Healthy Controls

The potential relationships between clinical characteristics and serum levels of MIP-3α and cystatin A are shown in Table 4. We found that patients with T3-4 tumor were more likely to have higher pretreatment MIP-3α levels than patients with T1-2 tumor (*P* = 0.011). Elevated cystatin A levels were more likely to appear in the older group (age ≥ 45 years) than in the younger group (*P* < 0.001).

**TABLE 4:**
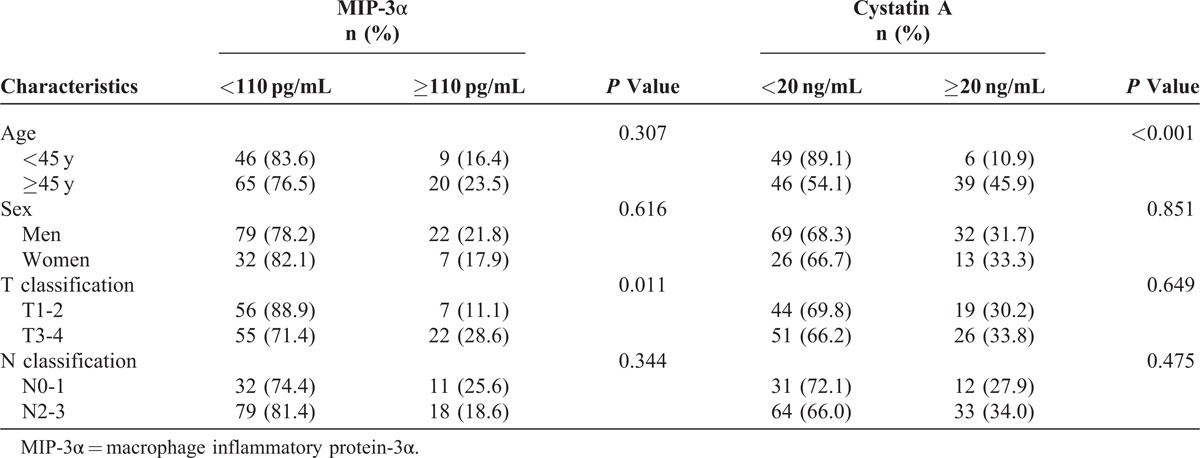
The Relationship Between Clinical Characteristics and Serum Levels of MIP-3α and Cystatin A

Spearman rank correlation analysis showed that pretreatment serum level of MIP-3α correlated weakly with cystatin A (*r* = 0.217, *P* = 0.010).

**FIGURE 1:**
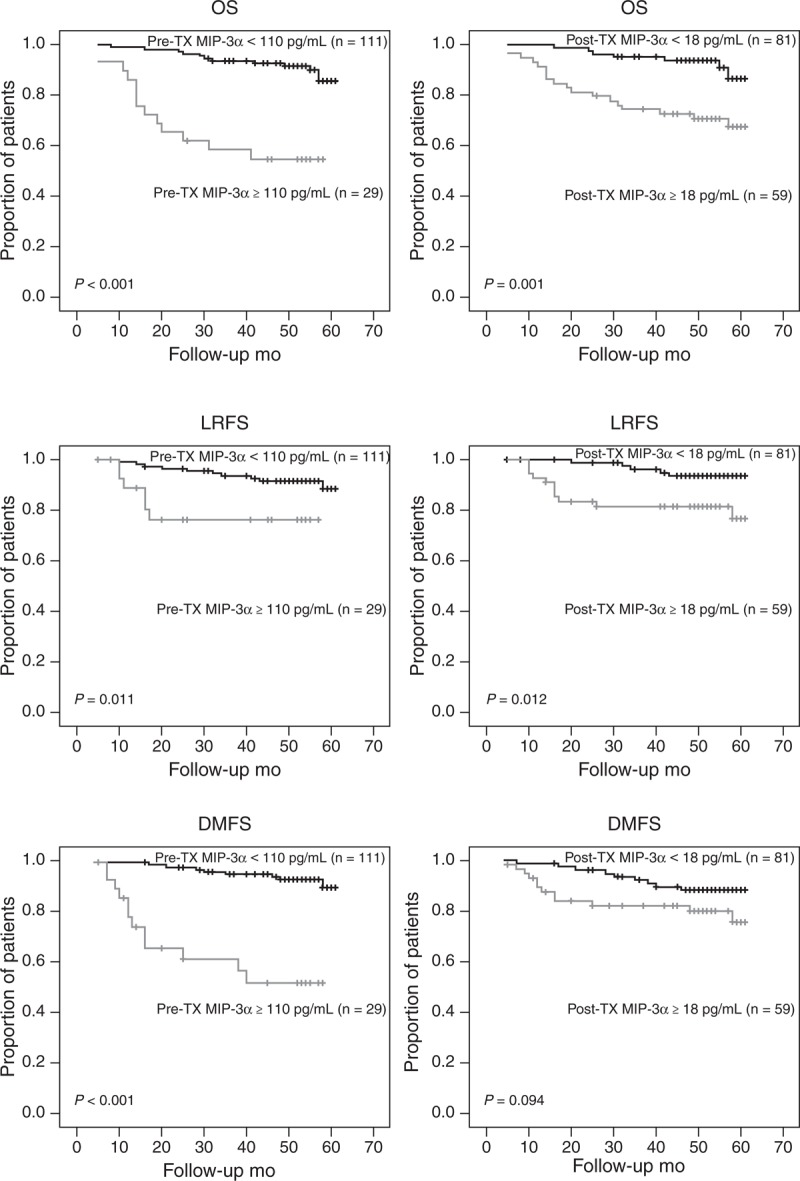
Kaplan–Meier plots of OS, LRFS, and DMFS in NPC patients according to different serum MIP-3α levels before and after treatment. DMFS = distant metastasis-free survival, LRFS = local recurrence-free survival, MIP-3α = macrophage inflammatory protein-3α, NPC = nasopharyngeal carcinoma, OS = overall survival, post-TX = posttreatment, pre-TX = pretreatment.

A cutoff value of 110 pg/mL (receiver operating characteristic curves constructed between death events and censors) was selected to categorize patients as high pretreatment MIP-3α and low pretreatment MIP-3α. Among all patients, 29 (20.7%) had higher pretreatment MIP-3α level (MIP-3α ≥ 110 pg/mL). The OS, LRFS, and DMFS curves for the high pretreatment MIP-3α level group and low MIP-3α level group are shown in Figure 1. Patients with high pretreatment MIP-3α level group had a poorer 4-year OS (OS, 54.8% vs 92.7%, *P* < 0.001), 4-year LRFS (LRFS, 76.2% vs 91.6%, *P* = 0.011), and 4-year DMFS (DMFS, 51.6% vs 92.3%, *P* < 0.001) than those with low pretreatment MIP-3α level group. Further, 59 (42.1%) had higher posttreatment MIP-3α level (MIP-3α ≥ 18 pg/mL). Patients with high posttreatment MIP-3α level had a poorer 4-year OS (OS, 72.7% vs 93.7%, *P* = 0.001) and 4-year LRFS (LRFS, 81.5% vs 93.5%, *P* = 0.012) than those with low posttreatment MIP-3α level; there was no statistical difference in 4-year DMFS curves between the 2 groups (79.9% vs 88.3%, *P* = 0.094) (Figure 1).

**FIGURE 2:**
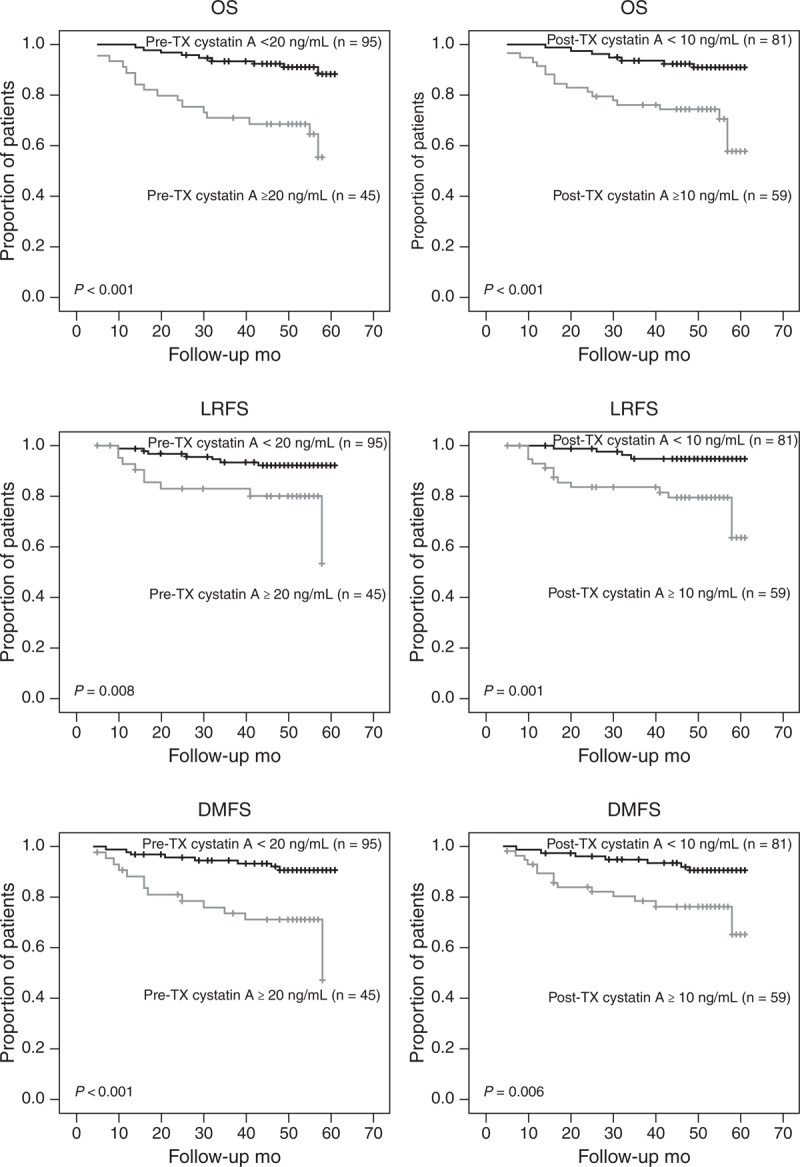
Kaplan–Meier curves for OS, LRFS, and DMFS in NPC patients according to different serum cystatin A levels before and after treatment. DMFS = distant metastasis-free survival, LRFS = local recurrence-free survival, NPC = nasopharyngeal carcinoma, OS = overall survival, post-TX = post-treatment, pre-TX = pretreatment.

A cutoff value of 20 ng/mL (receiver operating characteristic curves constructed between death events and censors) was selected to categorize patients as high pretreatment cystatin A and low pretreatment cystatin A. There were 45 (32.1%) patients with higher pretreatment cystatin A level (cystatin A ≥ 20 ng/mL). All the 4-year OS (OS, 55.3% vs 91.2%, *P* < 0.001), 4-year LRFS (LRFS, 80.3% vs 92.3%, *P* = 0.008), and 4-year DMFS (DMFS, 71.1% vs 90.8%, *P* < 0.001) of high pretreatment cystatin A level group were significantly lower than those of low pretreatment cystatin A level group (Figure 2). Moreover, there were 59 (42.1%) patients with higher posttreatment cystatin A level (cystatin A ≥ 10 ng/mL). All the 4-year OS (OS, 74.4% vs 92.5%, *P* < 0.001), 4-year LRFS (LRFS, 79.3% vs 94.9%, *P* = 0.001), and 4-year DMFS (DMFS, 76.4% vs 90.6%, *P* = 0.006) of high posttreatment cystatin A level group were also significantly lower than those of low posttreatment cystatin A level group (Figure 2).

The associations of pretreatment and posttreatment serum markers with OS, LRFS, and DMFS were examined further with Cox proportional hazards regression modeling, with adjustment for age, sex, WHO pathological classification, T classification, N classification, and chemotherapy. The analyses revealed that pretreatment serum MIP-3α and cystatin A levels were independent prognostic factors of OS and DMFS of NPC patients; serum posttreatment MIP-3α and cystatin A levels were independent prognostic factors of LRFS; and serum posttreatment MIP-3α level was also an independent prognostic factor of OS (Table 5).

**TABLE 5:**
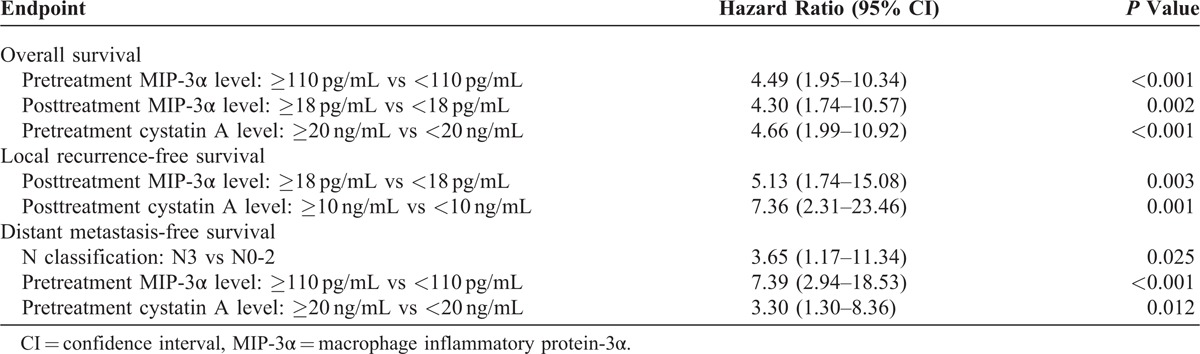
Multivariate Analysis of Prognostic Factors Using Cox Proportional Hazards Regression Model

## DISCUSSION

NPC is usually diagnosed at an advanced clinical stage, resulting in poor outcome. It is important to search new biomarkers that may be helpful for early diagnosis and prognosis prediction. With the development of molecular biology, several important biological markers have been identified to predict treatment response and outcome, and carry an important role in risk stratification and treatment selection.^13,14^

A study from Lau et al^15^ reported that infiltrating T lymphocytes may promote tumor growth and survival by membrane-binding ligands or cytokine secretion, and regulatory T-cells in tumor sites can enhance locally suppressive activity to help NPC cells evade antitumor immunity, but how T-cells are recruited to NPC tissues remains unclear. There is evidence that chemokines are produced by cancer cells and cells of tumor microenvironment including cancer-associated fibroblasts, mesenchymal stem cells, endothelial cells, tumor-associated macrophages, and tumor-associated neutrophils. Chemokines appear to promote tumor cell proliferation and metastasis by attracting endothelial cells, accelerating angiogenesis, or affecting the mobility of cancer cells.^16^ MIP-3α, encoded by the *CCL20* gene, is a strong attractant for T-cells and immature dendritic cells. Increased expression of MIP-3α has been reported in multiple myeloma^17^ and hepatocellular carcinoma.^18^ Our earlier studies have indicated that the detection of serum MIP-3α and cystatin A might contribute to improve the NPC staging and prediction of short-term clinical outcomes.^19^ In this study, by analyzing the relationship between MIP-3α and clinical characteristic prognostic factors, we found that patients with higher T-stage tumors were more likely to have increased levels of MIP-3α. Moreover, we found that serum MIP-3α concentration before treatment was a good indicator of posttreatment survival and distant metastasis in NPC patients; it was similar to the report by Chang et al.^9^ Significant decrease of MIP-3α after treatment implied that NPC cells were effectively cleared. Moreover, we also found that serum MIP-3α concentration after treatment was a good indicator of survival and local recurrence as well.

Basement membrane damage is regarded as an important indicator of tumor invasion. Moreover, the degradation of the extracellular matrix by proteolytic enzymes is a critical step of invasion and metastasis of tumor cells. Among the factors that promote tumor growth and invasion, several protease systems, which are involved in proteolytic degradation of extracellular matrix components, have been studied, including papain-like lysosomal cysteine proteases such as cathepsins B and L, as well as their endogenous inhibitors cystatins, for example cystatin A (also known as stefin A), cystatin B, and cystatin C.^8^ However, their roles in tumor progression are not clear yet. Cystatin’s inhibitory effect may either be beneficial to counteract tumor-associated proteolytic activity, or deleterious to suppress antitumor immune response. Cystatin A in nonsmall cell lung cancer,^20^ operable squamous cell carcinoma of the head and neck,^21^ and laryngeal cancer^22^ was upregulated; elevated level of cystatin A was positively correlated with better prognosis of patients. High serum cystatin A levels were also found in patients with hepatocellular carcinoma^23^ and colorectal cancer^24^; serum level of cystatin A was positively associated with tumor size and number of lesions of liver cancer, and patients with high serum cystatin A level had a poorer survival time in colorectal cancer. Upregulation of cystatin A in tumor tissues can reversely balance the overexpression of tumor-associated proteolytic enzyme activity in a certain range. Serum levels of cystatin A may reflect not only the changes in their local expression in tumors, but also a systemic immune response to malignant disease. Unlike the previous report,^10^ we found that pretreatment serum level of cystatin A was not associated with the nodal stage of NPC patients. Moreover, we demonstrated that serum cystatin A concentrations both before and after the treatment were good indicators not only of survival, but also of local recurrence and distant metastasis in NPC patients.

Our study suggests that serum MIP-3α and cystatin A can be used as new markers to define patients with a high risk of metastasis who warrant more aggressive systemic therapy to improve long-term outcome. Combining T and N stage with pretreatment and posttreatment MIP-3α and cystatin A levels may provide new clinical criteria of patient selection for combined modality treatment.

We found statistically significant correlation between MIP-3α and cystatin A. They may be intrinsically linked together to affect the pathogenesis and development of NPC. The specific mechanism still requires further research.

## CONCLUSIONS

Our results indicate that MIP-3α and cystatin A could serve as promising candidate markers in NPC prognosis. Higher serum levels of MIP-3α and cystatin A correlated with shorter probability of overall survival, local recurrence, and distant metastasis in univariate and multivariate analysis. However, a study of a large population is needed to evaluate the long-time prognostic importance and clinical relevance of these 2 factors in NPC.
